# Dual-Mode Sensing of Fe(III) Based on Etching Induced Modulation of Localized Surface Plasmon Resonance and Surface Enhanced Raman Spectroscopy

**DOI:** 10.3390/nano14181467

**Published:** 2024-09-10

**Authors:** Miriam Parmigiani, Benedetta Albini, Pietro Galinetto, Angelo Taglietti

**Affiliations:** 1 Department of Chemistry, University of Pavia, Viale Taramelli 12, 27100 Pavia, Italy; miriam.parmigiani01@universitadipavia.it; 2 Department of Physics, University of Pavia, Via Bassi 6, 27100 Pavia, Italy; benedetta.albini@unipv.it (B.A.); pietro.galinetto@unipv.it (P.G.)

**Keywords:** Localized Surface Plasmon Resonance (LSPR), Surface-Enhanced Raman Spectroscopy (SERS), iron (III) detection, oxidative etching, gold nanostars, silver

## Abstract

Convenient, rapid, highly sensitive and on-site iron determination is important for environmental safety and human health. We developed a sensing system for the detection of Fe(III) in water based on 7-mercapto-4-methylcoumarine (MMC)-stabilized silver-coated gold nanostars (GNS@Ag@MMC), exploiting a redox reaction between the Fe(III) cation and the silver shell of the nanoparticles, which causes a severe transformation of the nanomaterial structure, reverting it to pristine GNSs. This system works by simultaneously monitoring changes in the Localized Surface Plasmon Resonance (LSPR) and Surface-Enhanced Raman Spectroscopy (SERS) spectra as a function of added Fe(III). The proposed sensing system is able to detect the Fe(III) cation in the 1.0 × 10^−5^–1.5 × 10^−4^ M range, and its selectivity of the GNS@Ag@MMC sensor toward iron has been verified monitoring the LSPR and the SERS response to other cations with a clear selectivity toward Fe(III).

## 1. Introduction

Iron is one of the most important and common elements on earth. Iron cations play an essential role in a wide number of biochemical processes, and iron (III) is necessary in the human body and in all living organisms. On the other side, an excess of iron in the human body may result in damages or malfunctions of the heart, liver and pancreas, and it may produce damages to the central nervous system related to neurodegenerative disorders such as Alzheimer’s and Parkinson′s diseases [1,2]. Thus, iron concentration is a parameter of great importance to assess water environmental quality: the World Health Organization recommended 0.3 mg/L as the upper concentration limit of iron in drinking water. Since Fe(III) is the most abundant iron ionic species in the environment, its easy and rapid detection is of enormous importance [3,4].

To date, several probes and methodologies for the detection of iron (III) in solution were reported, ranging from atomic absorption spectrometry [5] to molecular and supramolecular fluorescent probes [6,7].

Iron sensing based on Carbon Dots (CDs) emission quenching, using different carbon sources, has become very popular, allowing to obtain sensing platforms which can detect the presence of iron(III) at milli-molar and even micro-molar concentrations [8].

Silver and gold nanoparticles (AgNPs and AuNPs) were also proposed for the detection of Fe(III) ions, mainly relying on their plasmonic features.

These kinds of assays can be based on the changes of Localized Surface Plasmon Resonance (LSPR) bands upon the aggregation of properly coated nanoparticles as a consequence of crosslinking caused by the interaction of Fe(III) cations with functional groups decorating their surfaces. In examples reported by Bothra et al. [9,10], the LSPR features of AgNPs, typically placed around 400 nm, disappear because of the aggregation and subsequent sedimentation of NP. As reported by Wu et al. [11], the aggregation of pyrophosphate-coated AuNPs takes place in the presence of Fe(III) ions, yielding a naked eye perceivable color change from pink to violet, which can be used to quantify ferric cations. In a recent example, biogenic AgNPs were used to detect Fe(III) ions using the same approach [12].

Another quite straightforward way to detect Fe(III) in solution using AgNPs is to exploit their oxidation given by the ferric cation. This strategy was successfully used by Chen et al. [13], who utilized N-acetyl-L-cysteine-stabilized silver nanoparticles. The redox reaction between Fe(III) and Ag(0) led to the formation of Fe(II) and Ag(I): the progressive etching of AgNPs causes the gradual depletion of the LSPR band, allowing Fe(III) detection. A similar approach was applied to detect Fe(III) cations using AgNPs prepared and stabilized using an extract of *Ficus retusa* [14]. 

Colorimetric approaches based on changes in the visible spectra can suffer from interference from colored analytes and, in this regard, additional and distinctive signals of the analyte of interest can be highly desirable [15]. In this sense, the use of Surface-Enhanced Raman Spectroscopy (SERS) can be easily coupled with a colorimetric approach based on LSPR. 

SERS has emerged as a powerful and widespread tool for sensing and analytical purposes, as it gives specific, well-defined, unique and easily recognizable vibration bands from the Raman spectra, coupled with a high sensitivity, which is ensured by the electromagnetic field coupled with nanostructured metal surfaces. SERS allows the rapid and real-time ultrasensitive detection of almost any analyte owing, generating or modulating a proper Raman active vibration [16,17]. 

Some examples appeared in the last few years proposing the SERS-based detection of iron cations. One way to use SERS for Fe(III) detection is to exploit the chelation of iron cations by siderophores like desferroxyiamine, properly attached to AgNP surfaces, bringing out the Raman modes relative to the Fe(III)-O bond at 580 and 1573 cm^−1^, which are considered markers of the chelation process [18,19]. 

Similar approaches were proposed using AgNPs decorated with other coating agents that are able to interact with Fe(III) and generate a change in Raman spectra revealed by SERS [20,21]. In a recent example [22], AgNPs coated with a cyanide-containing SERS probe were used to sense the presence of Fe(III). The authors exploited the interaction between the CN function and ferric cation, and subsequently the redox reaction of Fe(III) with Ag (0) previously described, and were able to monitor free iron and hemoglobin using LSPR and SERS signals. These examples clearly indicate that the synergy between LSPR and SERS increases the sensitivity, specificity, versatility and overall improved data reliability of the sensing approach [23,24,25].

On these bases, we decided to investigate a novel approach in Fe(III) detection with the use of the oxidative etching given by a ferric cation on a silver shell grown on a gold plasmonic core to obtain the modulation of LSPR spectra and of the SERS signals of a properly added Raman reporter. Both responses should be quantitatively dependent on the etching of the silver shell and thus from Fe(III) concentration. To achieve this, we used gold nanostars (GNSs) properly coated with a silver shell (GNS@Ag). 

GNSs are well-known effective SERS substrates [26,27], but when properly coated by a thin silver shell, a further boosting of SERS signals can be exploited [28,29,30]. Indeed, in a recent paper, we demonstrated how GNS@Ag with tips of GNSs still exposed outside of a properly optimized silver shell could yield a strong amplification of SERS signals compared to the one of pristine GNSs [31]. 

In this work, we used GNS@Ag NPs coated with 7-mercapto-4-methylcoumarine, which is typically used as excellent Raman reporter in SERS tags [27]. When Fe(III) is added to this colloidal suspension, oxidative etching can take place with a decrease in the shell thickness as a function of Fe(III) concentration. As GNS@Ag NPs revert to GNSs by the consumption of a silver shell, we expected a neat change in LSPR (observable by the naked eye or measurable by UV-Vis spectrophotometry) coupled with a neat decrease in SERS signals (measurable with a Raman spectrophotometer) of the MMC Raman reporter.

## 2. Materials and Methods

### 2.1. Chemicals

Tetrachloroauric acid (30% in HCl, 99.99%), 2-[4-(2,4,4-trimethylpentan-2-yl)phenoxy]ethanol (Triton-X-100), sodium borohydride (≥98%), ascorbic acid (≥99%), silver nitrate (≥99%), 7-mercapto-4-methylcoumarin (MMC) (≥97%), ethanol (≥99.8%), ammonium hydroxide solution (25%), iron (III) nitrate nonahydrate (≥99.95%), hydrochloric acid (≥37%), nitric acid (≥65%), magnesium sulfate (≥98%), calcium acetate monohydrate (≥99%), zinc acetate dehydrate (≥98%), cadmium acetate dehydrate (≥98%), manganese bis(trifluoromethanesulfonate), lead (II) acetate trihydrate, and iron (III) nitrate nonahydrate were purchased from Sigma Aldrich (St. Louis, MO, USA). Cobalt (II) nitrate hexahydrate was purchased from Honeywell Riedel-de Haën (Milan, Italy). Copper nitrate trihydrate was purchased from Supelco Analytical (Milan, Italy). Water was deionized and double distilled (ddH_2_O).

All the glassware used for nanoparticles was always cleaned with aqua regia (3:1 hydrochloric acid and nitric acid) for 15 min and then washed with double-distilled water three times for 3 min under sonication. In the end, the glassware was dried in an oven for 1 h at 120 °C.

### 2.2. Instruments

- Spectrophotometer: UV-Vis-NIR absorption spectra of colloidal suspensions, in the 300–1800 nm range, were taken with a Varian Cary 6000i spectrophotometer (Agilent, Santa Clara, CA, USA), while for the ones in the 300–1100 nm range, we used a Varian Cary 60 (Agilent, Santa Clara, CA, USA).
- Transmission Electron Microscopy (TEM): TEM images of the samples were collected on a Jeol JEM-1200 EX II instrument (JEOL Italia, Milan, Italy). To prepare the grids, 10 µL of the sample was dropped on nickel grids, 300 mesh, coated with a Parlodion membrane. The samples to be deposited were diluted with double-distilled water in different ways: colloidal GNSs were diluted 1:200; GNS@Ag@MMC were diluted 1:50; GNS@Ag@MMC with different quantities of Fe(III) were also diluted 1:50.
- Raman and SERS measurements were performed on the investigated samples using an automated and integrated confocal micro-Raman spectrometer, XploRA Plus HORIBA Scientific (Horiba, Ltd., Kyoto, Japan), equipped with an Olympus microscope BX43 (Horiba, Ltd., Kyoto, Japan). The microscope set-up consists of four different objectives with 10×, 50×, 50× with a long working distance and 100× magnification. The spectrometer is equipped with a motorized xy stage on which the investigated samples are positioned. The spectral resolution is about 1 cm^−1^. An Open Electrode CCD camera (Horiba, Ltd., Kyoto, Japan), with a multistage Peltier air-cooling system, is used as a detector. Two different solid-state laser sources have been used emitting light at 532 nm and 638 nm. The maximum power was 100 mW for green light and 90 mW for the red one. Neutral filters with different optical density allow for setting the incident laser power. The spectral resolution is about 1 cm^−1^; neutral filters with optical density equal to 2, 1, 0.6, 0.3 and 0 can be used to adapt the laser incident power, leading to power density values from 5 × 10^3^ W/cm^2^ to 5 × 10^5^ W/cm^2^.

### 2.3. Synthesis of Gold Nanostars (GNSs)

GNS were prepared following a well-known procedure previously described [32]. Seeds were prepared in a vial: first, we added 5 mL of HAuCl_4_ aqueous solution (4.50 × 10^−4^ M) to 5 mL of Triton-X-100 aqueous solution (0.2 M). Ultimately, 600 µL of an iced-cooled aqueous solution of NaBH_4_ (0.01 M) was quickly added to the previous solution: after this addition, the pale-yellow solution of AuCl_4_^−^ changed to a brown–orange color and was stored in an ice bath. Seeds must be used within 3 h. The growth solution was prepared starting from 50 mL of a water solution of Triton-X-100 (0.2 M), as for the seeds: then, we added, under magnetic stirring, 2500 µL of AgNO_3_ in water (0.004 M), 50 mL of aqueous HAuCl_4_ (4.50 × 10^−4^ M), 1700 µL of aqueous L-ascorbic acid (0.0788 M) and 120 µL of the seed solution previously prepared. After the last add, the suspension turns from pink to purple and then to blue, finally giving a gray colloid. The magnetic stirring was stopped 5 min after the last addition. GNSs were then characterized with UV-Vis-NIR spectra and TEM images.

### 2.4. Silver Coating of Gold Nanostars (GNS@Ag)

The silver shell around gold nanostars was made following the procedure reported in a previous work [31]: the optimal thickness of the silver shell was achieved by adding, to 30 mL of GNS solution, under magnetic stirring, 300 µL of AgNO_3_ (0.1 M) and 300 µL of ascorbic acid (0.1 M). The reduction of silver by ascorbic acid was initiated by the addition of NH_4_OH (60 µL). After only a few minutes, the color of the solution began to darken, turning from gray to brown.

GNSs were then characterized with UV-Vis-NIR spectra and TEM images.

### 2.5. Preparation of GNS and GNS@Ag Coated with 7-Mercapto-4-methylcoumarine

For the coating of GNS and GNS@Ag with 7-mercapto-4-methylcoumarin, we followed a well-known procedure reported in a previous work [27]. To a specific volume of GNS or GNS@Ag colloidal solution, a proper amount of stock solution of MMC (1.0 × 10^−3^ mol/L) was added to obtain a final thiol concentration of 5.0 × 10^−5^ mol/L. After one night of magnetic stirring, we recorded a UV-Vis absorption spectra of the coated GNS/GNS@Ag to verify the integrity of the sample. Then, GNS@Ag samples were centrifuged two times at 13,000 rpm, for 30 min. each time, to eliminate residual Triton-X-100 and MMC. The GNS samples, on the other hand, were centrifuged only once. 

In both cases, the supernatant was discarded and the precipitate was redeployed in the same starting volume. Again, another UV-Vis absorption spectrum of the coated nanoparticles was recorded in order to check again their stability and their integrity. 

GNS@MMC and GNS@Ag@MMC were also checked by TEM and Raman spectroscopy, searching for the main Raman peaks of MMC (reported in Table 1) [27,33,34,35].

### 2.6. Detection of Fe(III)

First of all, the GNS@Ag@MMC colloidal solution was diluted 1:10 with double-distilled water. Twelve 20 mL vials were prepared and 10 mL of the previously diluted colloidal solution was placed in them; subsequently, we added increasing amounts of stock solution of Fe(III) nitrate (1.0 × 10^−2^ mol/L) to each one so that we had final concentrations of Fe(III) ranging from 1.80 × 10^−5^ mol/L to 1.80 × 10^−4^ mol/L. At the end, we acquired UV-Vis-NIR and Raman spectra of the various samples, to follow, respectively, the absorption bands of nanoparticles and the main Raman peaks of MMC. UV-Vis spectra were acquired on 3 mL of sample as is without any further dilution. Raman spectra, on the other hand, were acquired on 1 mL, again without any further dilution. 

The titration of GNS@Ag@MMC with Fe(III) has been repeated three times on three different starting colloids.

### 2.7. Detection of Potentially Interfering Cations

Always working in a 20 mL vial with 10 mL of GNS@Ag@MMC diluted 1:10, we added a proper amount of potentially interfering cations in order to have a final concentration of 1.50 × 10^−4^ mol/L. The aim is to show that at the same concentration as iron, these other cations do not cause changes in GNS@Ag@MMC LSPR and SERS signals. As for iron, we acquired the UV-Vis and Raman spectra of these samples, using the same working conditions.

## 3. Results and Discussion

### 3.1. GNS and GNS@Ag Preparation, Characterization and Coating

**Figure 1:**
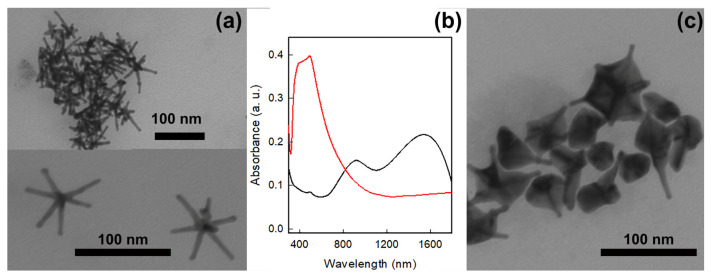
(**a**) TEM image of GNS; (**b**) UV-Vis-NIR spectra of GNS (black curve) and GNS@Ag (red curve); (**c**) TEM image of GNS@Ag.

GNS and GNS@Ag were prepared according to the described procedures [27,31,32,36]. TEM and UV-Vis spectroscopy were used for routine characterization. The expected morphology of GNS can be observed in Figure 1a, while in Figure 1b, the UV-Vis spectrum of a colloidal suspension of GNS is reported (black line), showing the expected LSPR band centered around 900 nm, due to the longitudinal resonance along a single branch. A second band can be found close to 1500 nm and is attributed to the longitudinal resonance involving two collinear branches.

The quantity of silver to be added in the shell preparation can be chosen in order to maximize the SERS enhancement, as recently demonstrated. The silver shell grows to the desired extent using GNSs as seeds and adding the proper quantity of silver ions in the presence of ammonia and ascorbic acid. As expected, the formation of the silver shell produces a neat spectral change that can be clearly perceived by the eye, as the deep blue suspension immediately turns to reddish–brown. The UV-vis spectrum of GNS@Ag is reported in Figure 1b (red line); Figure 1c shows the morphology of the GNS@Ag with the proper thickness of silver shell. The addition of Ag causes the disappearance of the LSPR bands typical of GNS, while a new LSPR band is present with a wide absorption maximum between 400 and 550 nm. In Figure 1c, a TEM image of GNS@Ag is reported. In this image, it is evident how original GNSs look almost completely coated with terminal parts of the branches protruding from the silver shell. The situation described was demonstrated to be the optimal to gain a maximum enhancement by using a silver shell around spiky gold nano-objects, as introduced by Vo-Dinh [28,29], and confirmed by previous experimental and theoretical work [31].

**Scheme 1:**
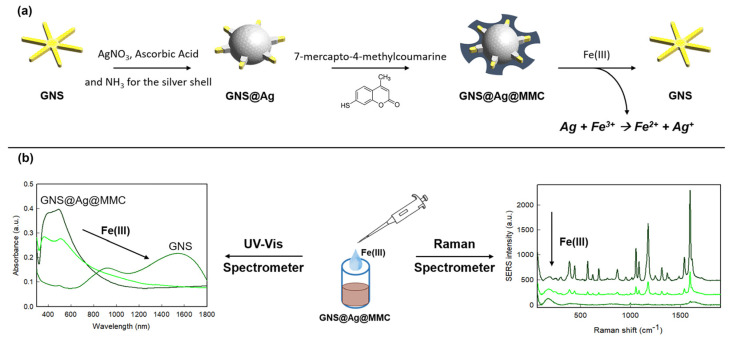
(**a**) Preparation process of the sensor, used materials and working mechanism; (**b**) experimental system of GNS@Ag@MMC-based detection.

At this point, objects were ready to be coated with a proper Raman reporter, i.e., a molecule with an intense and clearly recognizable Raman signal, coupled with the ability to stick closely to the nano-surface, which is a situation that maximizes the SERS response. We used 7-mercapto-4-methylcoumarin (MMC) because of its high Raman cross-section and of the thiol function, which allows the formation of a strong covalent bond with noble metals and is able to form a monolayer on the sample surface. Triton X-100 is weakly bound to GNS and GNS@Ag surfaces and thus can be easily removed with MMC. MMC was added as a stock solution to colloidal suspensions of GNS and GNS@Ag to reach a concentration of 5.0 × 10^−5^ M, which is known to ensure the formation of a monolayer on the nano-objects surface [27]. After one hour, colloidal suspensions were centrifuged and then redispersed twice in ddH_2_O to remove the surfactant, Raman reporter molecules in surplus (not grafted to nano-objects), and any other residual reactant. The preparation process and working mechanism of the sensing system are depicted in Scheme 1a; the experimental system is reported in Scheme 1b.

**Figure 2:**
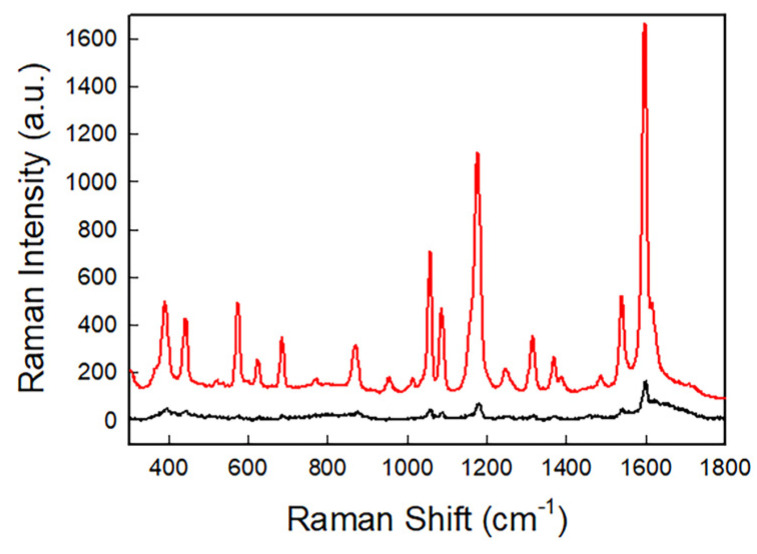
SERS spectra of GNS@MMC (black spectrum) and GNS@Ag@MMC (red spectrum).

Spectra of GNS@MMC and GNS@Ag@MMC do not differ substantially from those of the starting colloidal suspensions of GNS and GNS@Ag, and these are reported in Figure S1. The functionalization with MMC of GNS causes an increase in the local refractive index, leading to a moderate red shift (about 20 nm) compared to the UV-Vis spectra of pristine GNS (Figure S1) [37]. The SERS spectra of the re-dissolved suspensions confirm the effect of the silver layer, which can be appreciated in Figure 2. The typical SERS signature of MMC appears in both samples, with an evident enhancement for the GNS@Ag sample, with an amplification of more than one order of magnitude compared to the starting GNS.

### 3.2. Fe(III) Detection

**Figure 3:**
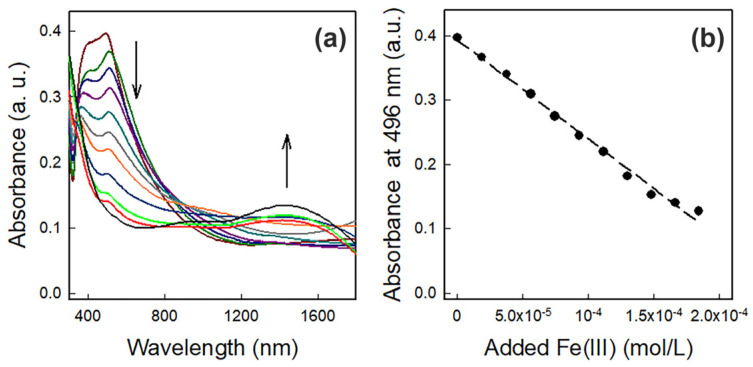
(**a**) The UV-Vis absorption spectra of GNS@Ag@MMC with increasing quantities of Fe(III); (**b**) maximum absorbance at 496 nm showing a linear trend vs. Fe(III) concentration.

In a typical experiment, a sample of the purified GNS@Ag@MMC colloidal suspension was diluted 1:10 with bi-distilled water. A series of three spectrophotometric titrations was carried on by adding increasing quantities of Fe(III) stock solution to different portions of the colloid. After an equilibration of 1 h, UV-Vis spectra of these samples with different Fe(III) concentrations were taken. As can be observed in Figure 3, which shows one representative experiment, the addition of an Fe(III) cation produced a decrease in the LSPR band centered between 400 and 550 nm with the linear profile evidenced in Figure 3b obtained plotting the extinction at 496 nm. Two others titrations (see Figure S2), along with their respective profiles at 496 nm, account for the reproducibility of the experiment. The profiles of the three titration allow to foresee a sensitivity around 1.0 × 10^−5^ mol/L and a working range that extends to 1.5 × 10^−4^ mol/L. It is of striking evidence how, during the titration, the plasmonic features in the 400–600 nm progressively decrease, a phenomenon attributable to silver shell etching, which is caused by the reaction reported in Equation 1.

Fe^3+^ + Ag^0^ → Fe^2+^ + Ag^+^ (1)

When the Fe(III) concentration reaches the value of 1.80 × 10^−4^ M, one can observe the presence of an LSPR band almost identical to the one of the starting GNS, suggesting that all the silver shell was etched by the added Fe(III).

**Figure 4:**
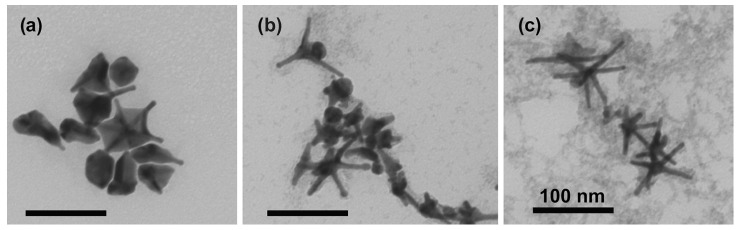
TEM images of (**a**) starting GNS@Ag@MMC; (**b**) GNS@Ag@MMC with 9.3 × 10^−5^ M Fe(III); (**c**) GNS@Ag@MMC with 1.8 × 10^−4^ M Fe(III), where it is possible to see the structure of the starting GNS.

TEM imaging confirms this hypothesis. In Figure 4, one TEM image of GNS@Ag@MMC (Figure 4a) is compared with the one obtained for the same sample in the presence of 9.30 × 10^−5^ M Fe(III) (Figure 4b) and with the one with a concentration of 1.80 × 10^−4^ M of Fe(III) (Figure 4c). As can be clearly observed, with the increase in Fe(III) concentration during the titration experiment, the silver shell is reduced but still present to a small extent (Figure 4b) at the middle of titration, while the shell is completely removed in Figure 4c, corresponding to the final point of titration shown in Figure 3. The GNS structure seems perfectly conserved, which is in good agreement with the spectra observed for the final point of titration. The presence of a granular gray substance in Figure 4b,c is due, reasonably, to the formation of silver and iron salts, which are formed during titration and, being soluble in the colloidal suspension, are then deposed on the TEM grid together with the nano-objects.

The same samples used for UV-Vis-NIR experiments were also tested as SERS substrates, following the SERS signals from MMC. We decided to monitor the SERS responses at two different wavelengths, 638 nm and 532 nm, studying the behavior at two different extinction values, with the green laser light closer to the plasmonic resonance band at 500 nm.

**Figure 5:**
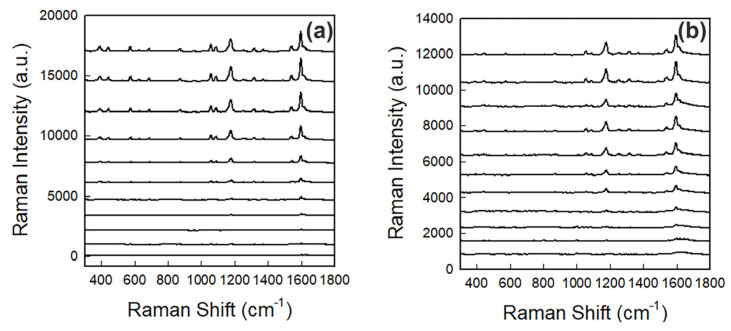
SERS spectra of GNS@Ag@MMC colloidal suspensions titrated with increasing quantities of Fe(III) (top–bottom) using laser wavelength at 638 nm (**a**) and 532 nm (**b**).

SERS spectra representative of the SERS titration experiments, both at 638 nm and 532 nm, are reported in Figure 5a,b, respectively.

**Figure 6:**
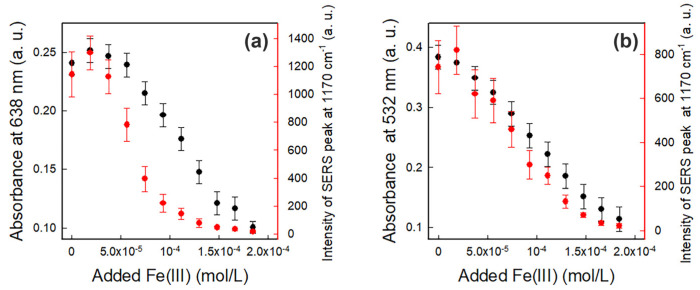
(**a**) Absorbance at 638 nm (black spots, left Y-axis) vs. SERS intensity at 1170 cm^−1^ peak (red spots, right Y-axis) excited at laser wavelength 638 nm. (**b**) Absorbance at 532 nm (black spots, left Y-axis) vs. SERS intensity at 1170 cm^−1^ peak (red spots, right Y-axis) excited at laser wavelength 532 nm.

For both laser excitation wavelengths, the expected depletion of SERS signals is reached at higher Fe(III) concentrations, but the observed dependence of SERS intensity vs. the amount of Fe(III) concentration is rather complex compared to what was previously found in the spectrophotometric measurements, obtained by plotting the value at 496 nm, which is the maximum of LSPR band intensity (Figure 3b). The SERS behavior as a function of Fe(III) concentration has been carefully analyzed following the intensities at 1170 cm^−1^, i.e., the signal due to the Raman mode associated to the -C-O stretching. These values have been compared to the UV-Vis spectrophotometric titration profiles using extinction values at 638 and 532 nm. The results are reported in Figure 6.

The comparison between SERS and extinction values at 638 nm indicates that both signals seem almost constant (likely a slight increase can be inferred) at the beginning of the titration, i.e., for Fe(III) concentrations lower than 5.0 × 10^−5^ M. At higher concentrations, the extinction value decreases almost linearly, while the SERS signal decreases sharply with a sizeable deviation from the linearity. Then, for SERS signals, a complete quenching (<5%) is registered as the Fe(III) concentration reaches the value of 1.50 × 10^−4^ M of Fe(III). The same analyses for SERS and extinction experiments at 532 nm indicate a more correlated behavior. Indeed, both values of SERS intensities at 1170 cm^−1^ and the extinction at 532 nm display an almost linear decrease as the Fe(III) amount increases. The above reported analyses point out that the SERS titration holds better at 532 nm. 

As already stated in the fundamental paper by Le Ru et al. [38], the connection between absorption and SERS enhancements can become more and more indirect in colloidal solution, i.e., in randomly distributed nano-objects, depending on different parameters such as for instance the nature, geometry and sizes of the nanostructure. This indirect relation depends in particular on the different role played in the two experiments by surface-like and bulk-like plasmon resonances. It is difficult to discern between different plasmonic contributions, but nevertheless, we can observe that using a 532 nm laser to measure the SERS response, the system is excited just close to the band maximum of plasmonic resonance (at around 500 nm). Even if some shifts occur that vary the Fe(III) concentration, it is reasonable to suppose that the mechanism involving plasmonic energy transfer between nano-objects and molecules does not change markedly, thus leaving the Fe(III) amount as the main driving parameter for SERS signals. On the contrary, when a red laser is used, we are exciting the system in a more complex plasmonic region where the overlapping of different plasmonic bands is appreciable. In this region, probably, varying the iron concentration, the role and weight of surface-like and bulk-like plasmon resonances are changing with a complex interplay that affects the linear behavior vs. iron concentration.

We underline that the plasmonic population in the reported experiments can vary greatly in terms of the nature and kind of plasmonic resonances. Indeed, the increase in Fe(III) causes a change in the shape of the nano-object, the gradual disappearance of Ag and the restoration of branched gold nanostars as an enhanced nanostructure. This observed behavior can be ascribed to the fact that SERS enhancement at 638 nm is probably due to higher-order resonances, while at 532 nm, closer to the extinction peak, a pure dipolar mode is acting. The marked changes in extinction, due to the main dipolar mode, can mask different changes in the higher-order phenomena when the Ag layer is markedly reduced, i.e., in the intermediate region [27].

The usage of SERS substrates as the Fe(III) sensor thus must be performed at a proper wavelength, depending on the particular set of nano-objects responsible for the filed enhancement. On the other hand, one can guess that with a proper design and a proper choice of laser wavelength (in this case 638 nm), the SERS substrate can act as a switch working with a digital logic, as suggested by the behavior reported in Figure 6a: a “small” change in Fe(III) concentration causes an abrupt decrease in the SERS signal with a sigmoidal profile. 

In order to further confirm the mechanism of the SERS response for Fe(III), we proceeded to additional investigations. Indeed, one cannot exclude the formation of a coordination complex between silver ions (released by the oxidation of silver shells) and the thiolate form of MMC. It is well known that strong coordinative interactions can be established between Ag(I) ions and thiolates [39], and thus the increase in Ag(I) concentration could result in the extraction of MMC from objects’ surfaces, which further contributes to the MMC signal decrease in SERS. To verify this, we proceeded to a UV-Vis spectrophotometric titration, where silver ions were added to a neutral (pH 6–7) water solution of MMC, with the same concentration present in the GNS@Ag@MMC colloidal suspensions used for Fe(III) determinations. MMC, in its molecular form, has a pKa of 3.4 in water. It has an absorption band close to 328 nm in strongly acidic solutions, while the anionic form, predominant for pH values higher than 3.4, shows an absorption close to 370 nm. What can be observed in Figure S3a is that the 370 nm band, typical of the thiolate anionic form of MMC present at the working pH, disappears when the Ag(I) concentration is increased with the formation of a band close to 330 nm. This band resembles the one owing to the S-H form, and in this case, it can be attributed to the presence of S-Ag(I) bonds in a complex between Ag(I) and deprotonated MMC (MMC^−^). Plots of the titration profiles at different wavelengths (Figure S3b) clearly indicate the formation of a 1:2 stoichiometry complex. These results corroborate the hypothesis that when Ag^+^ is formed upon oxidation of the silver shell of GNS@Ag@MMC caused by Fe(III), it can compete, as its concentration increases, for MMC binding with the surface of the nano-objects. This causes the SERS response to reach very low values before the silver shell is completely etched, as MMC^−^ (deprotonated anionic form of MMC) is removed from nano-object surfaces to form a complex with silver ions in solution. This is confirmed by the Raman spectra of a solution of Ag(MMC)_2_^−^ in the presence of a pristine GNS colloidal suspension compared with the SERS spectra obtained at the end of described titrations, the situation where the whole silver shell was removed, leaving the original GNS structure. As can be seen in Figure S4, the spectra are almost completely identical, again confirming the correctness of our interpretation: the completion of silver shell consumption leads to the formation of Ag(MMC)_2_^−^ with the removal of MMC^−^ from nano-objects surfaces, which is an event that, along with the change in plasmonic features of the same nano-object, leads to an almost complete depletion of SERS signals.

**Figure 7:**
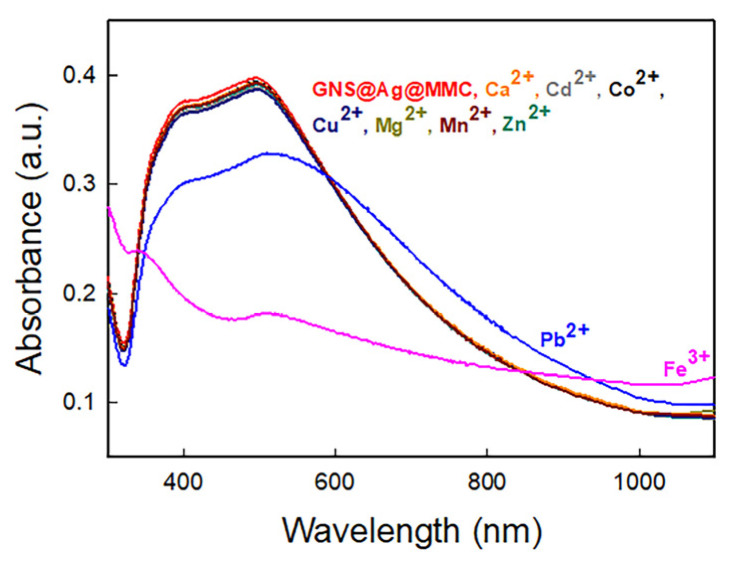
UV-Vis spectra of GNS@Ag@MMC with different cations at a concentration of 1.50 × 10^−4^ mol/L.

After having assessed the ability of the GNS@Ag@MMC system to reveal Fe(III) cations, we investigated the effects of other metal cations. Figure 7 reports the UV-Vis spectra obtained adding a final concentration of cations fixed at 1.50 × 10^−4^ M, and no effects are observed for all the cations investigated (Cu^2+^, Ca^2+^, Zn^2+^, Cd^2+^, Mg^2+^, Co^2+^, Mn^2+^). The only exception was Pb^2+^, where a decrease in the LSPR band of GNS@Ag@MMC was observed.

**Figure 8:**
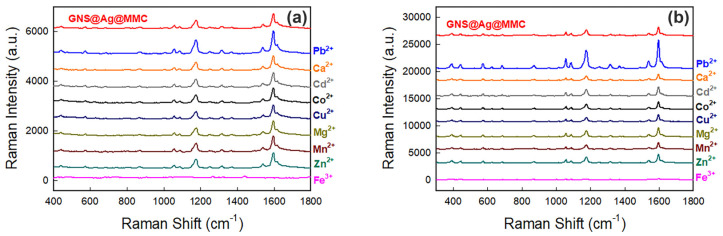
SERS spectra of GNS@Ag@MMC with different cations at a concentration of 1.50 × 10^−4^ mol/L. (**a**) Laser wavelength 532 nm; (**b**) laser wavelength 638 nm.

When the same measurements were repeated with SERS (Figure 8) with both the 638 nm and 532 nm lasers, none of the investigated cations gave SERS signal suppression as caused by Fe(III), except again for Pb^2+^, which produced a strong increase in Raman intensity. This increase is more evident with the 638 nm laser (Figure 8b) than with the one at 532 nm (Figure 8a). The peculiar behavior of Pb^2+^, both in the LSPR and SERS spectra, is probably due to the aggregation of GNS@Ag@MMC. This in turn can increase markedly the presence of hot-spots and nano-gaps between two, or more, nanoparticles. In this specific case, the gap distances can reach a few nanometers, becoming the source of very intense local electric fields [38] and thus a higher Enhancement Factor, and, at the same time, lowering and broadening LSPR bands [40]. This hypothesis has been confirmed also by TEM images of the samples: in Figure S5, it is possible to compare the starting GNS@Ag@MMC (a) with the samples with the same amount of Fe(III) (b) and Pb(II) (c). With Fe(III), as it has been said before, we can see that the silver shell around the gold nanostars is reduced and almost totally absent, whereas in the sample with Pb(II), the silver shell is still clearly visible, but a quite evident aggregation of the nano-objects can be noted. 

## 4. Conclusions

In this work, we confirmed how the deposition of a silver shell on a gold anisotropic nanostructure causes a strong enhancement in SERS signals of molecules located in the close surroundings. Based on this, we realized an experimental proof of concept for a sensing system selective for the Fe(III) cation. This cation triggers the removal of the silver shell on the nano-tags, which is a phenomenon that can be efficiently signaled both with LSPR and SERS techniques. A neat color change, from the brownish–pink of GNS@Ag@MMC to the light gray–blue of GNS, is indeed clearly perceptible with the naked eye, and it can be quantified by UV-Vis spectrophotometry. This was demonstrated upon the addition of Fe(III) to the colloidal suspension of the GNS@Ag@MMC system, showing a good linearity of the decrease in LSPR features close to 500 nm up to 1.50 × 10^−4^ M of Fe(III) concentration. Moreover, the two events connected with silver shell removal, i.e., the change in the plasmonic features of GNS@Ag and the removal of the Raman active capping ligand, allow for measuring the Fe(III) concentration also by means of SERS. The naked eye perception, the quick response and the possibility of dual-mode sensing make this system an easy-to-use and trustable method to detect the ferric cation in solution with simple instrumentations (UV-Vis spectrophotometer and Raman apparatus), and it represents an alternative to more complex and time-consuming options. The SERS responses at red and green excitations exhibit different behaviors, linear and mainly sigmoidal respectively, suggesting the possibility of using different lights for different sensing logics. We thus believe this work can give some contribution to a new class of detection systems based on the modulation of plasmonic and SERS [41] features of properly designed structures.

## Figures and Tables

**Table 1:** Main Raman modes of MMC.

Peak Position (cm^−1^)	Assignment
438	Skeletal vibration
570	Ring deformation
685	Skeletal vibration and -C-O deformation
1057	Characteristic ring vibration
1102	Characteristic ring vibration
1170	-C-O- Stretching
1318	-C-O- Stretching
1366	Conjugated C=C- asymmetric stretching and -C-H deformation
1384	-C-CH_3_ deformation
1544	Stretching -C-C-, -CH_3_ deformation and -C-H*_ring_* in-plane deformation
1593	Conjugated -C=C-symmetric stretching

## Data Availability

Data is contained within the article and supplementary material.

## Supplementary Materials

The following supporting information can be downloaded at https://www.mdpi.com/article/10.3390/nano14181467/s1, Figure S1: Comparison of UV-Vis spectra of GNS and GNS@Ag before and after coating with MMC; Figure S2: Repetition of LSPR titrations (300–1100 nm) of GNS@Ag@MMC with Fe(III); Figure S3: Titration of MMC with increasing quantities of silver ions. Figure S4: SERS spectra of Ag(MMC)_2_^−^ vs. GNS@Ag@MMC at the end of the titration. Figure S5: TEM images of GNS@Ag@MMC with Fe(III) and Pb(II).
