# Supplementary material for: Dual-Mode Sensing of Fe(III) Based on Etching Induced Modulation of Localized Surface Plasmon Resonance and Surface Enhanced Raman Spectroscopy
Source: Nanomaterials (Basel). 2024 Sep 10;14(18):1467. doi: 10.3390/nano14181467 (PMC11434494; doi:10.3390/nano14181467)
Supplement: Supplementary file 1 [file nanomaterials-14-01467-s001.zip › nanomaterials-3082478-supplementary.pdf]

Supplementary Material

Dual-Mode Sensing of Fe(III) Based on Etching Induced Modulation of Localized Surface Plasmon Resonance and Surface Enhanced Raman Spectroscopy

Miriam Parmigiani [1], Benedetta Albini [2], Pietro Galinetto [2] and Angelo Taglietti [1,*]

[1] Department of Chemistry, University of Pavia, Viale Taramelli 12, 27100 Pavia, Italy; miriam.parmigiani01@universitadipavia.it
[2] Department of Physics, University of Pavia, Via Bassi 6, 27100 Pavia, Italy; benedetta.albini@unipv.it (B.A.); pietro.galinetto@unipv.it (P.G.)
* Correspondence: angelo.taglietti@unipv.it

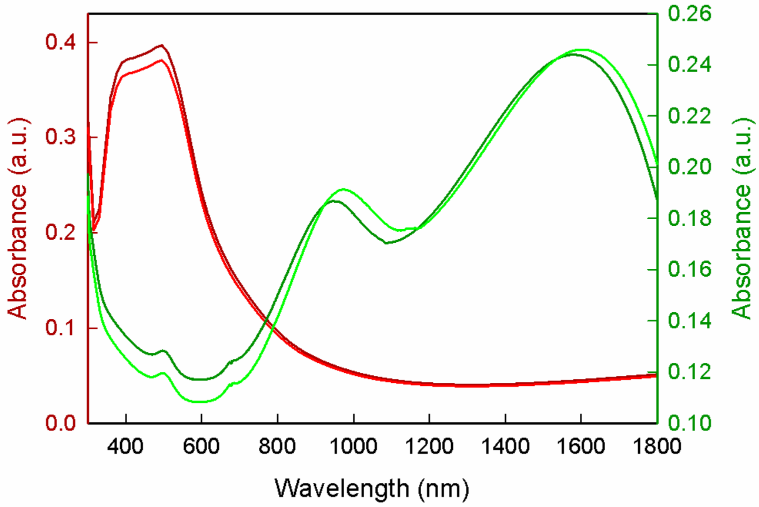

Figure S1. Additional comparison between UV-Vis spectra reported in Figure 1b and Figure 2a, about the coating of GNS and GNS@Ag with MMC, with the corresponding red-shifts. The red curves are GNS@Ag (darker curve) and GNS@Ag@MMC (lighter one), with reference to the left Y-axis; the green one instead are GNS (lighter curve) and GNS@MMC (darker curve), with reference to the right Y-axis.

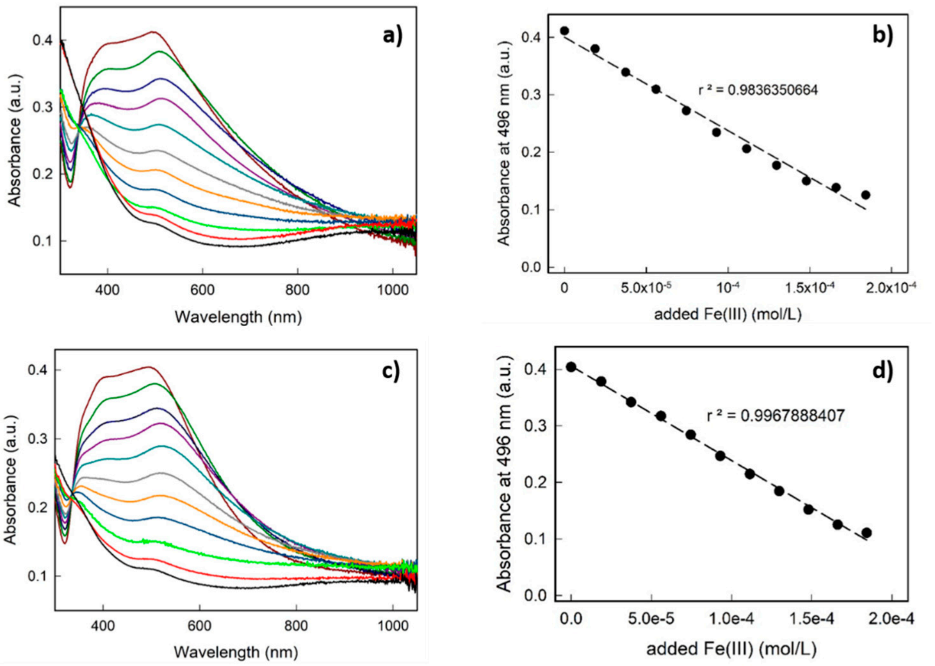

Figure S2. a) and b) Second experiment with UV-Vis aborption spectra (300 -1100 nm) of GNS@Ag@MMC with Fe(III), with maximum absorbance at 496 nm; c) and d) Third experiment and its 496 nm profile.

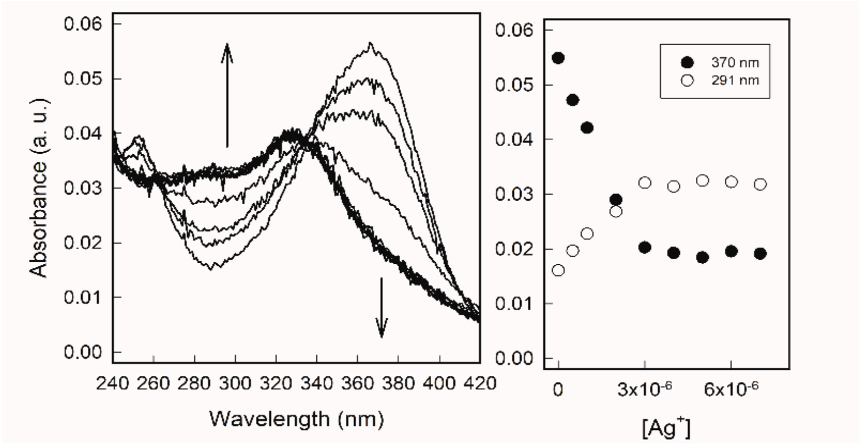

(a) (b)

Figure S3. (**a**) Titration of MMC (5 x 10^{-6} mol/L) with increasing quantities of silver ions (from $AgNO_3$); (**b**) LSPR profiles at 370 nm and 291 nm plotted against Ag^+ concentration.

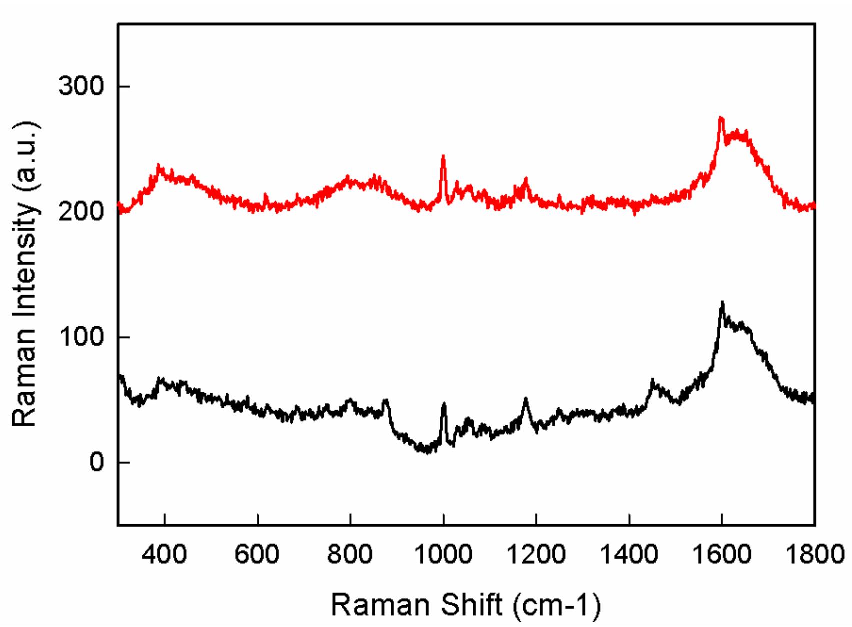

Figure S4. SERS spectra of $Ag(MMC)_2^-$ complex in GNS colloidal solution (black curve) compared to the one of GNS@Ag@MMC at the end of the Fe(III) titration (Fe(III) = 1.8 x 10^{-4} mol/L) (red curve).

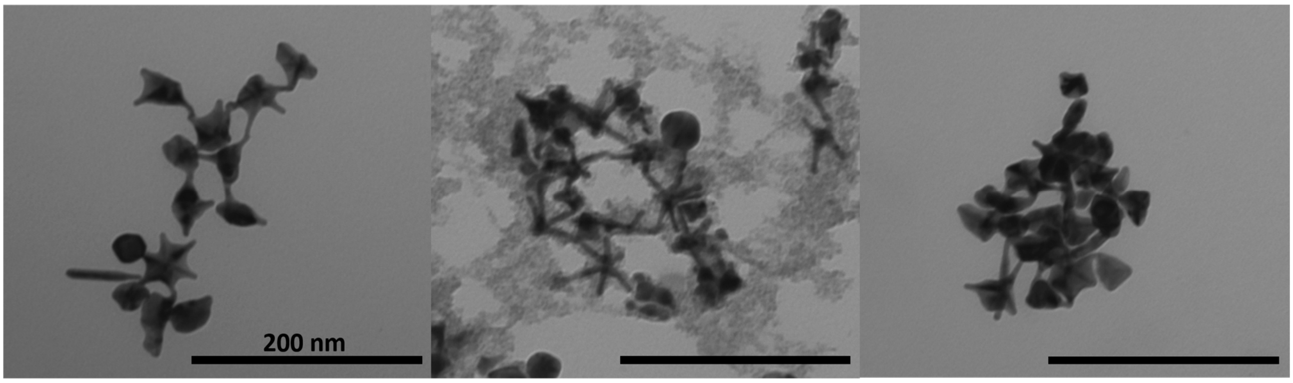

(a) (b) (c)

Figure S5. TEM images of (**a**) GNS@Ag@MMC; (**b**) GNS@Ag@MMC after being treated with a concentration of Fe(III) of 1.50 x 10^{-4} mol/L; (**c**) GNS@Ag@MMC after being treted with a concentration of Pb(II) of 1.50 x 10^{-4} mol/L.
